# Comparative Analysis of Cell Mixtures Deconvolution and Gene Signatures Generated for Blood, Immune and Cancer Cells

**DOI:** 10.3390/ijms241310765

**Published:** 2023-06-28

**Authors:** Natalia Alonso-Moreda, Alberto Berral-González, Enrique De La Rosa, Oscar González-Velasco, José Manuel Sánchez-Santos, Javier De Las Rivas

**Affiliations:** 1 Cancer Research Center (CiC-IBMCC, CSIC/USAL & IBSAL), Consejo Superior de Investigaciones Científicas (CSIC), University of Salamanca (USAL), & Instituto de Investigación Biomédica de Salamanca (IBSAL), 37007 Salamanca, Spain; nataliaalonsom@usal.es (N.A.-M.); aberralgonzalez@usal.es (A.B.-G.); enriquedlrm98@usal.es (E.D.L.R.); oscargv@usal.es (O.G.-V.); jose@usal.es (J.M.S.-S.); 2 Division of Applied Bioinformatics, German Cancer Research Center (DKFZ), 69120 Heidelberg, Germany; 3 Department of Statistics, University of Salamanca (USAL), 37008 Salamanca, Spain

**Keywords:** cell mixture, deconvolution, immune cells, blood cells, gene signature, bioinformatics

## Abstract

In the last two decades, many detailed full transcriptomic studies on complex biological samples have been published and included in large gene expression repositories. These studies primarily provide a bulk expression signal for each sample, including multiple cell-types mixed within the global signal. The cellular heterogeneity in these mixtures does not allow the activity of specific genes in specific cell types to be identified. Therefore, inferring relative cellular composition is a very powerful tool to achieve a more accurate molecular profiling of complex biological samples. In recent decades, computational techniques have been developed to solve this problem by applying deconvolution methods, designed to decompose cell mixtures into their cellular components and calculate the relative proportions of these elements. Some of them only calculate the cell proportions (supervised methods), while other deconvolution algorithms can also identify the gene signatures specific for each cell type (unsupervised methods). In these work, five deconvolution methods (CIBERSORT, FARDEEP, DECONICA, LINSEED and ABIS) were implemented and used to analyze blood and immune cells, and also cancer cells, in complex mixture samples (using three bulk expression datasets). Our study provides three analytical tools (corrplots, cell-signature plots and bar-mixture plots) that allow a thorough comparative analysis of the cell mixture data. The work indicates that CIBERSORT is a robust method optimized for the identification of immune cell-types, but not as efficient in the identification of cancer cells. We also found that LINSEED is a very powerful unsupervised method that provides precise and specific gene signatures for each of the main immune cell types tested: neutrophils and monocytes (of the myeloid lineage), B-cells, NK cells and T-cells (of the lymphoid lineage), and also for cancer cells.

## 1. Introduction

### 1.1. Cell Heterogeneity

The transcriptome analysis, as a global profile of gene expression, is a key factor for the study of complex biological samples composed of multiple cell populations in heterogeneous mixtures. Specifically, transcriptomics allows us to identify how genes change according to the biological processes happening in the human organism, or under specific pathological alterations that modify cellular function, such as tumorigenesis and cancer development. For example, increased infiltration of pro-inflammatory immune cells (primarily CD8+ cytotoxic T cells) in the tumor microenvironment (TME) is associated with a good prognosis in cancer patients [1,2,3,4], whereas the presence of immunosuppressive cells, such as myeloid-derived suppressor cells (MDSCs), regulatory T cells, tumor-associated macrophages (TAMs) and fibroblasts have an adverse effect, reducing the efficacy in oncological treatments [5,6,7]. These studies are usually based on the quantitative analysis of global gene expression (bulk RNA signal), obtained using different techniques that measure total RNA levels (normally mRNA). In this work, we used global gene expression data collected using microarray technology and RNA sequencing (RNA-seq). The microarray techniques are cheaper and require less time to run the algorithms than RNA-seq. However, this technology is limited to known genes or sequences inserted into the microarrays. RNA-seq is computationally more complex but allows the identification of new genes by measuring any expressed sequence and also detects genes with lower expression levels [8].

Using these global gene expression technologies, massive RNA data has been produced for millions of samples in thousands of transcriptomic studies over the past few decades, including highly relevant and accurate information on gene activity. The identification of the role of specific cells in bulk RNA data generally requires the application of specific experimental techniques, such as flow cytometry or immuno-histochemistry, which have major limitations since they are expensive, time consuming and usually restricted to known available markers, and to the possibility of separating and isolating the cells [9]. In contrast, computational techniques for deconvolution of cell mixtures do not have all these limitations and can be applied to decompose bulk signals, infer the relative frequencies of different cells contained in a sample, and also identify marker genes associated with specific cell types [9,10].

### 1.2. Deconvolution to Decompose Mixtures

Global signals or numerical values are generally used to measure all elements present as a mixture in a complex sample (e.g., a biological sample composed of multiple cell types). These mixture samples are decomposed using a mathematical method to identify their elements and calculate the number of components and their relative composition or percentage. Deconvolution is the mathematical term for this type of analytical approach. Usually, to explain this mathematical procedure, the phenomenon called the ‘cocktail party problem’ is used [11]. The experiment entails recording many people present at a party with many microphones, with the aim of disaggregating the voices and identifying a particular auditory stimulus by filtering and eliminating the rest of the voices [11,12]. Bringing this concept to biological omics data, and to massive transcriptomics data from complex biological samples, when global gene expression profiling data is collected using full transcriptomics (either with high-density microarray technology or with deep sequencing RNA-seq), an overall signal is obtained (bulk signal) that is made up of a mixture of signals and can be decomposed by applying deconvolution methods. In this case, the expression signal of each gene would be a cocktail, and the microphones that collect the signal would be represented by the samples present in the bulk [13].

### 1.3. Formulation of Deconvolution

Peng Lu, Aleksey Nakorchevskiy and Adward M. Macotte were the first to use such methodologies, estimating the quantity of distinct yeast cell types at various stages of the cell cycle [14]. Since this time, many deconvolution methods have been developed [10,15,16,17,18,19,20,21], and probably the most practical and successful application has been in blood samples (which include many different cell types) and in tissue samples infiltrated with blood and immune cells. Deconvolution algorithms decompose a mixture of different cell types into their constituent elements and calculate their proportion or ratio and, in some cases, also calculate the overall expression signal of the factors or features (i.e., genes). Let n, m and c be the number of genes, samples and cell types, respectively. Global or bulk transcriptomic data can be defined as follows:

(1) $$B_{nxm}=S_{nxc}*P_{cxm}$$

where $B_{nxm}$ is the mixture expression matrix (or bulk), $S_{nxc}$ is the signature matrix (i.e., the matrix of genes that mark the expression of c cell types), and $P_{cxm}$ is the proportion matrix (i.e., the data matrix which contains the relative frequencies of cell types in the mixed samples m). For the deconvolution process to be successful, the P matrix must fulfill two properties: (i) the columns (samples) must sum 1 ($\sum_{j=1}^{m}P_{kj}=1,\forall k\in \left[1,\ldots ,c\right]$); (ii) each element of the matrix must have a value between 0 and 1 ($0\leq \sum_{j=1}^{m}P_{kj}⩽1;\forall k\in \left[1,\ldots ,c\right],\forall j\in \left[1,\ldots ,m\right]$).

This global data B can also be explained as a set of equations, one per gene for each of the samples (in total n × m), where the value b_ij_ is a linear combination of the expression level s_ik_ of gene *i* (*i* = 1…*n*) in cell type *k* (*k* = 1…*c*), weighted by the proportion p_kj_ of cell type k in sample j [6]. Therefore, for each fixed sample j (j = 1…m), the model is formulated as follows:

(2) $$\left\{\begin{array}{c} b_{1j}=s_{11}p_{1j}+s_{12}p_{2j}+\ldots +s_{1c}p_{cj}\\ b_{2j}=s_{21}p_{1j}+s_{22}p_{2j}+\ldots +s_{2c}p_{cj}\\ \ldots \\ b_{nj}=s_{n1}p_{1j}+s_{n2}p_{2j}+\ldots +s_{nc}p_{cj} \end{array}\right.$$

There are two types of deconvolution methods depending on the elements to be estimated. When the aim is only estimating one of the two matrices (S or P), the method is known as partial deconvolution (supervised methods) and requires, in addition to a mixture matrix B, another remaining matrix (S or P) that provides the gene signatures or cell proportions. However, if the method can infer both matrices, so it only needs the B matrix, then it is a complete deconvolution, and the algorithm is defined as an unsupervised method [22]. In our work, we implemented three supervised methods: CIBERSORT, FARDEEP and ABIS; and two unsupervised methods: LINSEED and DECONICA. Within this methodological framework, this study has two main goals: (i) perform a comparative analysis of the results obtained with different cell mixture deconvolution methods; (ii) apply these methods to a series of complex mixtures of blood, immune and cancer cells, for which the proportions of cell types have been previously determined experimentally (in this way, we know the cellular composition a priori). Thus, the objective is not only to assess the accuracy in estimating cell proportions, but also to evaluate the identification of biological markers (i.e., gene signatures) that best separate the investigated cell mixtures.

## 2. Results

### 2.1. Comparison of Cell Type Proportions Correlations Using Four Deconvolution Methods

**Figure 1:**
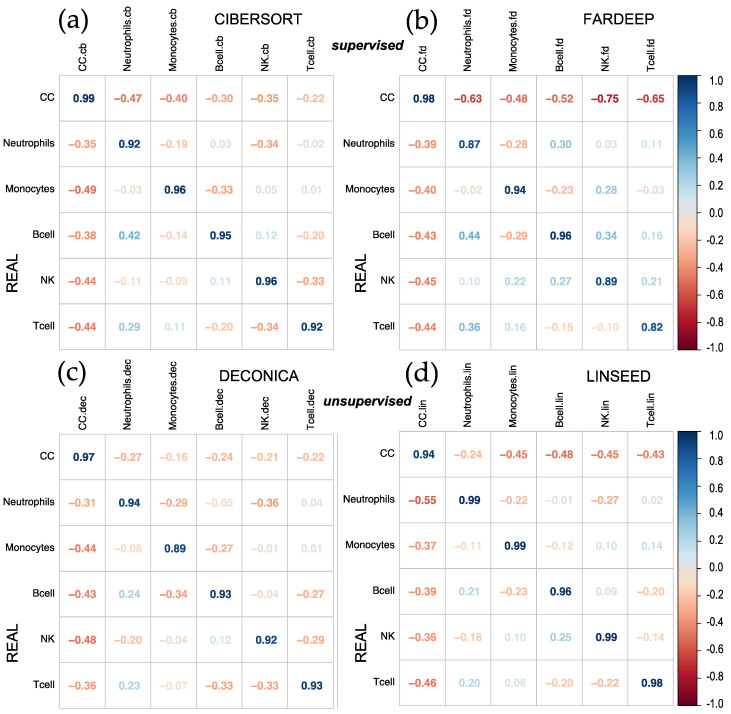
Corrplots comparing real versus estimated cell proportions. Pearson correlations were calculated with the 12 samples of the dataset (GSE64385), between the real proportions (rows) and the estimated proportions (columns) obtained with 4 methods: (**a**) CIBERSORT, (**b**) FARDEEP, (**c**) DECONICA and (**d**) LINSEED. The samples included 6 cell types mixed in known proportions: Cancer Cells (CC), Neutrophils, Monocytes, B cells, NK cells and T cells. GSE64385 includes the bulk gene expression data used in the deconvolution analyses.

First, we analyzed the results obtained after the implementation of CIBERSORT, FARDEEP, DECONICA and LINSEED, using a dataset (GSE64385) that includes purified cell populations, mixed in known proportions. To evaluate the different algorithms, a correlation plot (corrplot) has been made for each method. In the first case, shown in Figure 1, the proportions of tumor cells HCT116 (Cancer Cells, CC) and five immune cells were analyzed: Natural Killer cells (NKs), B lymphocytes (B cells), neutrophils, T lymphocytes (T cells) and monocytes. The highest correlation coefficients were obtained for the proportions calculated by CIBERSORT and FARDEEP. In addition, the unsupervised methods (DECONICA and LINSEED) also showed high correlation values between the estimated and real cell proportions, as indicated by the calculated correlation coefficients. In fact, LINSEED showed the best average correlation (= 0.975), improving the second best average correlation (= 0.95) obtained with CIBERSORT. As a whole, the actual or real proportions of each type of cell in the samples (real and estimated) are not revealed in the corrplots, so this way of analyzing and representing the data is not optimal since it does not present critical information about the relative amount of different cells included in a mixture. 

**Figure 2:**
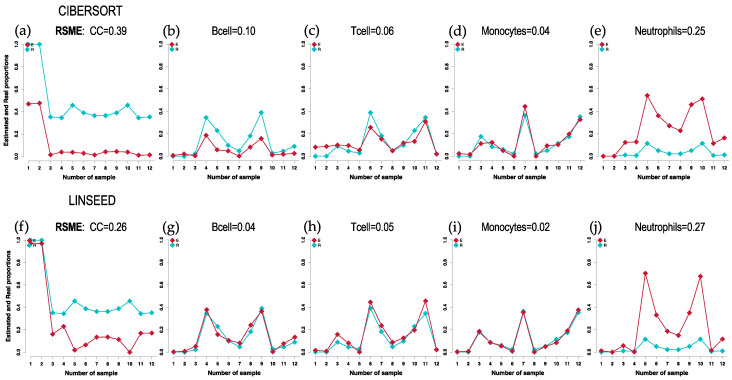
Cell-signature plots obtained for 3 cell types: Cancer Cells (CC), B-cells and T-cells, Monocytes and Neutrophils; using GSE64385 dataset. The plots include in blue the real proportions (Real) of each cell type in each of the 12 samples (marked with squared dots) and in red the estimated proportions (Estimated). The cellular signatures obtained with 2 different methods are presented: for CIBERSORT (**a**–**e**); and for LINSEED (**f**–**j**). The RSME (Root Mean Square Error) calculated between the real data (blue) and the estimated data (red) is presented at the top of each graph.

Therefore, other plots (cell-signature plots and bar-mixture plots) were created. The cell-signature plots for CIBERSORT and LINSEED are presented in Figure 2, showing a generally good estimation of the different cells proportions. The estimated CC (red dots) are lower than the real (blue dots) in the case of CIBERSORT, and they are lower in LINSEED only when the CC were mixed with other immune cells. The estimated CC are equal to real in the case of LINSEED when there were only cancer cells (Figure 2f). In fact, LINSEED is the method that shows the lowest Root Mean Square Error for estimation of cancer cells (0.26) and also for B-cells, T-cells and Monocytes (RMSE = 0.04, 0.05 and 0.02, respectively). Supplementary Figure S1 includes the cell-signature plots corresponding to the results obtained with DECONICA and FARDEED for CC, B-cell, T-cell and Monocytes. These two methods compared to CIBERSORT present much worse performance (i.e., higher RMSE) in estimating the proportions of the different cell types.

**Figure 3:**
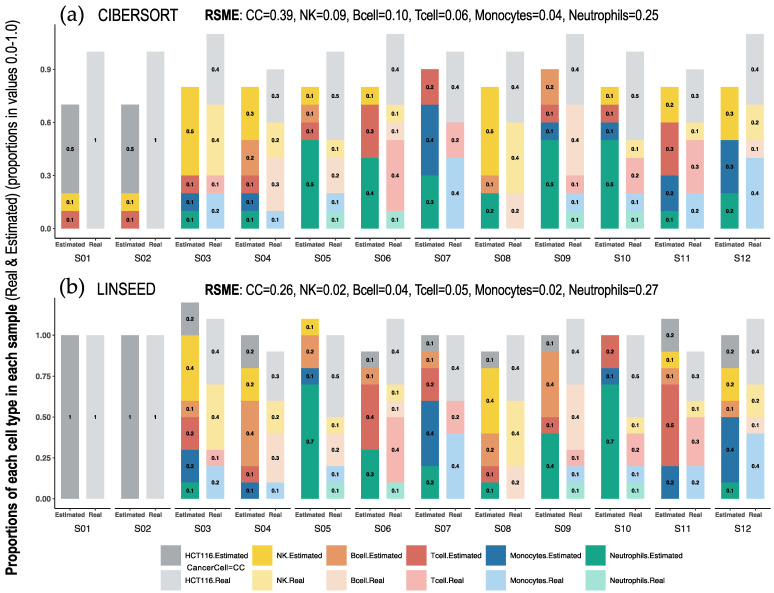
Bar-mixture plots. Bar plots presenting the cell mixtures in each sample as proportional sections of each cell type, which are marked with the colors presented in the color panel at the bottom of the figure. The estimated proportions in each sample were calculated with (**a**) CIBERSORT and (**b**) LINSEED. The real proportions in each sample (determined experimentally by flow cytometry) are presented as bars on the right in pale colors. The first sample (S01) only includes Cancer Cells (100% HCT116 cells). The RMSEs (Root Mean Square Errors) calculated between the real data and the estimated data for each cell type, are presented at the top of each graph.

On the other hand, Figure 3 shows the dissimilarity between CIBERSORT and LINSEED regarding the cell prediction when noise is present in the data. In this case, the noise is represented by tumor cells, which are found 100% in the first two samples (pure cancer cell samples), of which we had no marker genes in the immune signature matrix used by CIBERSORT. The abundance of immune cells inferred by the methods for the first and second samples would be zero, because these are composed exclusively of malignant cells, and consequently, the fractions of immune cells obtained by flow cytometry are zero. Despite this, CIBERSORT overestimated the values of some cell populations (monocytes and B and T lymphocytes) and did not estimate any CC in the remaining samples, which were composed of a mixture of tumor and immune cells.

This comparative analysis shows that LINSEED was able to recognize the presence of cancer cells in all samples, even when the proportion of these cells was lower (such as 0.3, 30%, in S04). Meanwhile, CIBERSORT found cancer cells in the samples S01 and S02 (which were composed of pure cancer cells, 100%) but could not find any cancer cells in the rest of the samples (samples S03 to S12, where the actual proportions were 30%, 40% or 50%). These results demonstrated the great capacity of LINSEED to calculate the proportions of cancer cells and the fact that CIBERSORT is a method focused mainly on immune cells (Figure 3). In this way, both analytical views (Figure 2 and Figure 3) are complementary and provide a comprehensive visualization of cell type proportions in the sample mixtures studied. Finally, we also include in Supplementary Figure S2 additional bar-mixture plots for the DECONICA and FARDEED methods (to complement the view presented in Figure 3). The results with DECONICA (Supplementary Figures S1 and S2), showed a poor performance with low variability in the cellular composition of the different samples; that is, all estimated proportions are distributed around a mean value.

### 2.2. Comparison of Proportions of 17 Cell Types, Identified in PBMCs, Calculated Using Different Deconvolution Methods against the Proportions Experimentally Determined

In this section, three supervised deconvolution methods (CIBERSORT, FARDEEP and ABIS) were applied to calculate a large collection of cell types and subtypes identified in PBMCs.

**Figure 4:**
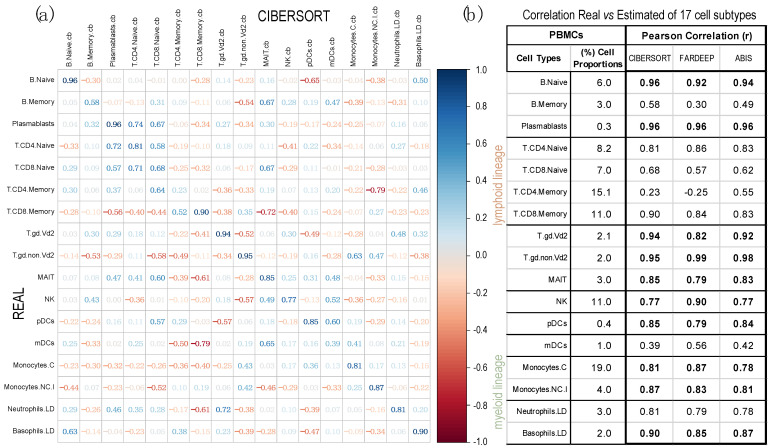
Correlations obtained with the gene expression profiles from 13 PBMC samples (taken from dataset GSE107011), calculated using 3 deconvolution methods. Pearson correlations were calculated between the real proportions (rows) and the estimated proportions (columns) for 17 cell types and subtypes (12 from the lymphoid lineage and 5 from the myeloid lineage). The correlations were calculated using: (**a**) CIBERSORT (corrplot) and (**b**) FARDEEP and ABIS (see table; in this case, only the diagonal values of real versus estimated for each cell type are included). (Labels of the cells: B.Naive = B Cells Naïve; B.Memory = B Cells Memory; Plasmablasts; T.CD4.Naive = CD4+ T Cells Naive; T.CD8.Naive = CD8+ T Cells Naive; T.CD4.Memory = CD4+ T Cells Memory; T.CD8.Naive = CD8+ T Cells Naive; T.gd.Vd2 = γδ2+ T Cells; T.gd.non.Vd2 = γδ2− T Cells; MAIT = Mucosal-Associated Invariant T Cells; NK = Natural Killer Cells; pDCs = Plasmacytoid Dendritic Cells; mDCs = Myeloid Dendritic Cells; Monocytes.C = Classical Monocytes; Monocytes.NC.I = Non-Classical Intermediate Monocytes; Neutrophils.LD = Low-Density Neutrophils; Basophils.LD = Los-Density Basophils.) The real mean proportions of the cells in the 13 PBMC samples, determined experimentally, are indicated (in %) in the second column of the table (**b**).

The analyses were performed using 13 PBMC samples, obtained from dataset GSE107011. For these samples, we had global gene expression data (i.e., the full transcriptomic profiling determined by RNA-seq), plus the proportions of each cell type in each sample determined experimentally. Figure 4 presents the Pearson correlations between the cell proportions calculated by each method and the real proportion of each cell type. The data show that CIBERSORT is the best method with an average correlation of 0.78, presenting the worse correlation for: Memory B-cells (0.58), Memory T-cells CD4+ (0.23) and myeloid Dendritic Cells (mDC, 0.39). The other methods are not better than this. In fact, not considering these three very specific cell types, CIBERSORT shows the best average correlation of 0.86, revealing a quite correct adjustment to the real cellular concentrations. FARDEEP and ABIS show both a mean correlation of 0.84, for the same 14 cell types. Supplementary Figure S3 presents the corrplots of these two methods.

### 2.3. Identification of Cell-Specific Gene Signatures Obtained by the Combination of Two Deconvolution Methods

**Figure 5:**
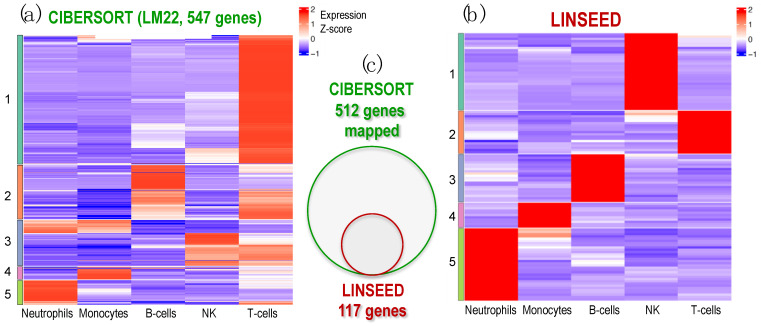
Heatmap of expression profiles corresponding to the genes selected in the signature matrices provided by CIBERSORT and LINSEED for 5 major cell types: Neutrophils, Monocytes, B-cells, NK cells and T-cells. The analysis was performed using dataset GSE64385. (**a**) Heatmap presenting the expression profiles of the LM22 data matrix, provided by CIBERSORT platform, which includes 547 genes used to identify the 5 cell types tested. (**b**) Heatmap presenting the expression profiles of the 117 genes selected by LINSEED (unsupervised method) from the gene list provided in the LM22 matrix (i.e., the same used by CIBERSORT). (**c**) Venn diagram presenting the genes that each method uses in the signatures to identify the 5 cell types in dataset GSE64385. All the genes selected by LINSEED are included in the ones used by CIBERSORT. The set of 117 genes, selected by LINSEED, provides more precise and specific gene signatures for each of the 5 cell types analyzed.

Finally, an analysis of cell signatures was performed to identify five immune cells (T and B lymphocytes, NKs, neutrophils and monocytes), provided by LM22 matrix (used in CIBERSORT and FARDEEP) and by those estimated by the unsupervised methods LINSEED, considering the matching genes between LM22 signature matrix and these ones present in the GSE64385 expression data (512 genes). For this purpose, we chose to apply a clustering analysis, whose results are shown in Figure 5 through the expression level of the marker genes for each cell type.

The analysis in search for gene signatures for the cells carried out with LINSEED was quite specific, since by using the LM22 cell data matrix provided by CIBERSORT, we were able to select with this unsupervised method 117 genes as cell markers for the 5 cells studied. Supplementary Figure S4 presents the set of genes selected as gene signature for each cell type, which are: 21 genes for B-cells; 19 genes for T-cells; 34 genes for NK cells; 11 genes for Monocytes; and 32 genes for Neutrophils. These gene signatures are much more specific that the ones included in LM22. In this way, LINSEED is an unsupervised method that can optimize the biomarkers proposed by the authors of CIBERSORT (https://cibersortx.stanford.edu/, accessed 10 June 2022), decreasing the number of genes contained in the signature matrix while maintaining the most important genes, which are well reported in the literature. All the data corresponding to these two heatmaps are included in the Supplementary Materials as: Supplementary Table S1 (this includes the genes presented in the heatmap in Figure 5a produced using CIBERSORT, with 547 genes arranged in order as in the heatmap clusters); Supplementary Table S2 (this includes the normalized expression signal of the 547 genes presented in the heatmap of Figure 5a); Supplementary Table S3 (this includes the genes presented in the heatmap in Figure 5b produced using LINSEED with 117 genes arranged in order as in the heatmap clusters); and Supplementary Table S4 (this includes the normalized expression signal of the 117 genes presented in the heatmap of Figure 5b).

## 3. Discussion

The precise analysis of the cellular composition and heterogeneity of complex biological samples opens the way to identify cellular marker genes, to determine changes in specific biological processes due to different cells, as well as to analyze the initiation and development of pathological states or diseases, driven by certain cells in a given organism. Previous studies in human tumors have demonstrated the clinical impact of the infiltration of certain immune cells, especially T lymphocytes [23,24], and the influence of the relative abundance of stromal cells, particularly adipocytes or fibroblasts, which can be related to tumor progression, invasion, metastasis, or drug resistance [25,26,27,28]. However, experimental techniques to identify the specific cell types present in a complex biological sample, such as flow cytometry or immunohistochemistry, are limited by the ability to separate cells and by the presence of known phenotypic markers. Therefore, analysis of the cellular composition of biological samples using deconvolution computational approaches capable of decomposing complex signals from cell mixtures (i.e., bulk signal) into specific cellular proportions, as well as identifying specific cell biomarkers, is a powerful approach that is rapidly evolving.

In our work, we implemented five deconvolution methods: three supervised (CIBERSORT, FARDEEP, and ABIS) and two unsupervised (DECONICA and LINSEED) using global expression signal (bulk) data (GSE64385, GSE106898 and GSE107011). In general, the most accurate methods were CIBERSORT and LINSEED, with high correlations, low RMSE values and cell distributions as the real known data. Both methods performed best using the LM22 matrix as the signature matrix (which, as mentioned above, is a cell-signature matrix developed by the creators of CIBERSORT). Given that the calculation of cell proportions is directly related to the signatures considered and that most of the methods use mathematical regression models, they are expected to have similar results. However, for the supervised methods to be accurate in their computations, the signature matrix and the bulk data must be collected by the same expression platform, as Chen et al. mention in their article [29]. In addition, CIBERSORT is one of the most widely used deconvolution methods nowadays [30,31,32,33,34], providing good results in predicting cell abundance. Nevertheless, LINSEED was more robust in the presence of noise in the data (i.e., in the presence of cancer cells). The FARDEED method [35], using the same LM22 as CIBERSORT, removing outliers before deconvolution and avoiding the alteration of the original data matrix, did not performed well.

Regarding DECONICA, the frequencies calculated may create confusion about its accuracy. The general correlation coefficients were high, and furthermore, the RMSE values did not differ much from the errors calculated for CIBERSORT. However, DECONICA cell proportions were distributed around a mean value, so there is no variability between the calculated proportions for the samples. Based on this fact, it is recommended to use LINSEED, instead of DECONICA, when the signature array is not available (i.e., in the case of unsupervised methods). In addition, when the bulk expression signal had a large number of genes, the computation of gene pairwise correlations was very time consuming, further reducing the accuracy of DECONICA. With respect to the use of ABIS, it was necessary to know the real proportions, which were used to calculate the scale factor, before running the algorithm. Therefore, if the objective is only to use the method to estimate cell abundance, it is not recommended to use it, because an experimental determination of the cell proportions would have to be applied previously. Regarding cell signatures, LINSEED was a powerful tool to optimize the sets of cell marker genes provided by CIBERSORT, selecting a much reduced number of marker genes contained in the LM22 reference matrix (designed to identify 22 immune cell types).

The RMSE values obtained for the deconvoluted expression data used in Section 2.2 were lower than those calculated for the expression data used in the first part (Section 2.1). This could overestimate the precision of these analyses performed over 17 cell types and subtypes. However, these lower RMSE values relate to the presence of smaller proportions of cell types. A clear example of this is the case of dendritic cells (DC) or plasmablasts, whose actual proportions are normally distributed below the 1% range. However, NK cells or monocytes are usually above 10%. So, the RMSE measurement is not comparable when the cell types present very different concentrations (or proportions) in the bulk.

In general, from our study, we can state that none of the studied methods for cell mixture deconvolution is adaptable to address all possible circumstances in the analysis of expression profiles obtained from complex biological samples, and a robust comparative analysis of the results is essential to assess the value and capacity of each method. In this context, it is important to note that the aim of this paper was not to present a “benchmarking” of deconvolution methods, but rather a study in which we compare the results of several well-known first-generation deconvolution approaches to obtain gene signatures and to facilitate the identification of major immune cells and cancer cells. In recent years, a large number of publications have addressed the benchmarking of deconvolution methods. From 2019 to 2022, several relevant benchmark and comparative studies of deconvolution methods were reported: in 2019, Sturm et al. published a comprehensive evaluation of transcriptome-based cell-type quantification methods applied to estimate nine different immune and stromal cells in bulk RNA-seq samples [36]; then, in 2020, Avila Cobos et al. published a highly cited article presenting a benchmarking of cell type deconvolution pipelines for transcriptomics data [37]; and in 2021, Jin and Lui presented another updated benchmark for RNA-seq deconvolution analysis comparing 11 popular deconvolution methods [38]; finally, in 2022, Sutton et al. published a comprehensive evaluation of deconvolution methods comparing eight transcriptome deconvolution approaches and nine cell type signatures, testing the accuracy of deconvolution using single-cell RNA-seq and RNA mixtures data [39]. Overall, a recent review article presents an overview of 20 deconvolution techniques showing the clear expansion of this field of research in recent years [40].

Considering these recent comparative studies, we are well aware of the existence of novel second-generation deconvolution methods, such as: CIBERSORTx [41], MuSiC [42] and DWLS [43] (all published in 2019) and other more recent ones, such as: BISQUE [44] (2020), and SCDC [45] (2022). All these methods are considered “second generation” because they are based on the use of single-cell experimental information for the deconvolution procedure. In this work, we avoid these new-generation methods because we focus on first-generation deconvolution methods that do not use a priori single-cell information, and can be: supervised methods (if they required to calculate the cell proportions the preliminary information from a gene signature matrix that allows the identification of cell types: CIBERSORT, FARDEEP and ABIS); and unsupervised methods (if they infer both the signature matrix and the cell proportions: LINSEED and DECONICA).

Despite this great development in deconvolution procedures, there are still important problems for the accuracy of these methods that are mainly associated with the quality of the reference data used. The analysis of cell mixtures in real biological samples faces serious difficulties associated with: sample heterogeneity and variability, sample purity and cross-contamination, overlapping of cell signatures, technical noise from cell determination methods, batch effects, etc. These problems need to be addressed both by improving cell-specific determination technologies (for example, by achieving a true-omics coverage of single-cell techniques, or by avoiding bias towards certain cells due to different forms and shape or to alterations during isolation); and by the improvement of computational developments (for example, with better methods to integrate multiple data sources, or with the application of powerful artificial intelligence (AI) algorithms such as Deep Neural Networks (DNNs)).

A major biological problem in cellular deconvolution is the lack of specific gene signatures for all the different cell types, which causes the “spillover effect”: an overlap of gene signatures between different cell populations [36,39,46]. In particular, in the case of immune cells, there is frequent confusion in some myeloid cells (such as neutrophils, dendritic cells or macrophages subpopulations), which are difficult to identify within a complex mix of cellular signals, especially if they come from different organs or tissues. In this respect, a future challenge to improve the decomposition of cell mixtures is the generation of accurate and highly specific gene signatures for each possible cell type in different biological contexts (for example, not only for many immune cells, but also for many tumoral cells that can have a very different origin and nature). The results obtained in this work with LINSEED, namely, the identification of cancer cells and the reduction in the gene signature for immune cells, is a clear positive step in this direction.

## 4. Materials and Methods

### 4.1. Datasets

We used for the analyses three cell mixture datasets, two of them including genome-wide expression data obtained using high-density microarrays technology: GSE64385 [10] and GSE106898 [47]; and another one obtained using RNA-seq technology: GSE107011 [48]. These datasets are composed of peripheral blood mononuclear cells (PBMCs) or polymorphonuclear cells (PMNs), and one of them (GSE64385) also includes human colon cancer cell line HCT116 [10] in the mixture (being in this set two samples of pure cancer cells, and the others known amounts of different immune cells plus cancer cells). In the case of dataset GSE107011, 13 PBMC samples were selected for analysis from a total of 127 (these samples were as follows: GSM2859500, CYFZ_PBMC_rep9; GSM2859501, FY2H_PBMC_rep8; GSM2859502, FLWA_PBMC_rep10; GSM2859503, 453W_PBMC_rep5; GSM2859504, 684C_PBMC_rep6; GSM2859505, CZJE_PBMC_rep7; GSM2859531, DZQV_PBMC_rep4; GSM2859532, 925L_PBMC_rep2; GSM2859533, 9JD4_PBMC_rep1; GSM2859534, G4YW_PBMC_rep3; GSM2859535, 4DUY_PBMC_rep11; GSM2859536, 36TS_PBMC _rep12; GSM2859537, CR3L_PBMC_rep13). Information about the specific cell types that compose the mixtures in each dataset can be found in Supplementary Figure S5. A summary with information about each dataset is presented in Table 1.

Supervised methods require a gene signature matrix, which must contain the expression profiles of the gene markers used to identify the different cell types (i.e., one gene signature for each cell type interrogated in the sample mixture). These gene signature matrices are usually generated using the same platform as the analyzed samples. In our analysis, we used three gene signatures matrices (all of these data matrices present the genes as rows and the cell types as columns):

(i) LM22: Signature matrix composed of 22 immune cell types and 547 genes, designed by CIBERSORT authors [10]. We used it to decompose the mixture samples (bulk expression data) of dataset GSE64385.
(ii) ‘sigmatrixMicro.txt’: Matrix consisting of 819 genes characterizing 11 immune cell types in complex cell mixtures. Signal expression was obtained with Illumina microarrays [47]. We applied this matrix to decompose the bulk in GSE106898.
(iii) ‘sigmatrixRNAseq.txt’: Signature matrix composed of 1296 gene biomarkers to identify 17 immune cell populations. Signal expression was obtained with Illumina RNA-seq [48]. We applied this matrix to deconvolute the bulk in GSE107011.

### 4.2. Brief Description of the Cell Mixture Deconvolution Methods Used

#### 4.2.1. DECONICA: Deconvolution through Immune Component Analysis

This is an unsupervised deconvolution method and therefore only requires the mixture expression matrix or bulk [13]. For the estimation of cell types, it is based on the algorithm FastICA [49], which uses a multivariate technique (ICA: Independent Component Analysis) whose objective is to find uncorrelated latent variables, which present a non-Gaussian distribution (with skewness and kurtosis coefficients maximized far from zero). The aim is to obtain a matrix *A* (representing absolute frequencies of the cell types in the samples) whose numbers maximize the skewness and kurtosis statistics of the distribution. Therefore, with n being the number of observable variables (genes), m the number of samples, and c the components into which to decompose the data, the mixture expression matrix (B) can be formulated as the product of matrix A and the signature matrix S, as shown by Equation (3).

(3) $$B_{nxm}={S_{nxc}*A}_{cxm}$$

#### 4.2.2. LINSEED: Linear Subspace Identification for Gene Expression

This method, like the previous one, solves a complete deconvolution since it is also an unsupervised method. In this case, cell type-specific genes are defined by their exclusive expression in only one component within a mixture. In an ideal scenario, the gene markers expression behaves exactly linearly with the proportions of the corresponding mixture component. Therefore, expression levels of the biomarkers to the same mixture component are also mutually linear with each other. Subsequently, to deconvolute a mixture of signals, LINSEED identifies the marker genes (the specific genes) for each cell type, by calculating the mutual linearity between pairs of genes [50]. Mutual linearity of cell type-specific genes suggests that the space of the mixed gene expression profiles might have a distinct underlying structure. Thus, the method systematically investigates the topological properties of a common space that can be generated from two related space matrices: matrix X defines an expression space with genes as dimensions and samples as data points; and matrix H defines a proportions space with cell types as dimension and samples as data points [15]. The rows of both matrices, H and X, have the same dimensionality (equal to the number of samples in the dataset, m). This means that the vectors that make up the transposed matrices H^T^ and X^T^ have the same dimensionality and can be mapped as points within the common m-dimensional space. These mapping and transformation steps are performed using the algorithm Simplex [51], which facilitates the convergence of row-normalized vectors of expression and cell proportion visualized in this m-dimensional space, in which the vertices (i.e., the corners of a multi-dimensional hyperplane representing the optimal points) are the cell types, and the closest points to each vertex their specific gene markers. In mathematical terms, the problem is formulated as follows:

(4) $$\mathrm{Max}\;(\min)\;z:\;{\tilde{X}}_{i}^{T}=\sum_{j}^{c}\alpha_{j}^{i}{\tilde{H}}_{j}^{T};\;\;\forall_{i}\alpha \geq 0\wedge \sum_{j}^{c}\alpha_{j}^{i}=1$$

where $\tilde{X}$ is the mixture expression matrix (like B in previous methods, row-normalized per gene), $\tilde{H}$ is the cell type proportions matrix (like P matrix, row-normalized per cell type), and α is a non-negative coefficient, which must sum to one per sample.

#### 4.2.3. ABIS: ABsolute Immune Signal Deconvolution

It is a supervised method that can be applied to decompose the whole gene expression data [48]. Before deconvolution, this method requires normalization by mRNA abundance, providing an optimal α coefficient for each cell type, which allows the difference between estimated and actual values to be calculated. The mathematical formula for α is defined as:

(5) $$\underset{\hat{\alpha }\epsilon \left(l,u\right)}{min}\sqrt{\sum_{i=1}^{c}{{(\hat{p}}_{i}-p_{i})}^{2}}$$

Subsequently, the expression of the signature matrix (per cell type) is multiplied by this coefficient, and the deconvolution is performed. For deconvolution, ABIS is based on a robust linear model (RLM), which for each gene and sample is described by Equation (6).

(6) $$b_{i}={\hat{p}}_{1}{{\hat{\alpha }}_{1}s}_{i1}+{\hat{p}}_{2}{{\hat{\alpha }}_{2}s}_{21}+\cdots {\hat{p}}_{c}{{\hat{\alpha }}_{c}s}_{ic}+\varepsilon$$

Considering n the total gene number and c the cell types to be estimated. For any gene i (∀ i∈[1,…,n]) and any cell type k (∀ k∈[1,…,c]), $b_{i}$ is the expression of the gene in the bulk, ${\hat{p}}_{k}$ is the cell type proportion, ${\hat{\alpha }}_{k}$ is the mRNA abundance and $s_{ik}$ represents the expression of the gene i into the corresponding cell type k.

#### 4.2.4. FARDEEP: Fast and Robust Deconvolution of Expression Profiles

This is a supervised method designed to solve partial deconvolution problems, previously eliminating outliers that may disrupt the results. For this purpose, FARDEEP is based on the aLTS (Adaptive Least Trimmed Squares) algorithm [52], which incorporates outliers:

(7) $$b_{i}={\hat{p}}_{1}s_{i1}+{\hat{p}}_{2}s_{i2}+\cdots {\hat{p}}_{c}s_{ic}+\tau +\varepsilon$$

where *τ* = (*τ*_1_ + *τ*_2_ + ⋯ + *τ*_n_)^T^ indicates that the i-th gene (*i* ∈ [1,…, *n*]) is an outlier. For more information regarding the outliers estimation, see the original article [35].

#### 4.2.5. CIBERSORT: Estimation of Cell Types Abundances in a Mixed Cell Population Using Gene Expression Data

Supervised method that solves a partial deconvolution, so mixture and signature matrices are needed as parameters. To perform deconvolution, it is based on the machine learning algorithm known as Support Vector Regression (SVR) [8], which is a feature of Support Vector Machine (SVM). This algorithm represents the regression model that best fits the data on a hyperplane, selecting support vectors (in our case, the support vectors are the marker genes) that define the limits of the error (*ε*) that the model can tolerate [26]. The hyperplane is defined by Equation (8):

(8) $$MIN\frac{1}{2}{\left\|p\right\|}^{2}+C\sum_{i=1}^{n}\left|\varepsilon_{i}\right|$$

where p represents the proportions of the cell types to be estimated, and C is a positive constant that allows us to control the error, so if this value increases, then the tolerance for points outside *ε* will also increase. Finally, $\varepsilon_{i}$ is the parameter that controls the error determined by the support bands (defined by the support vectors), calculating the distance between the points represented outside them and the limits of the acceptance region.

## 5. Conclusions

In summary, one of the main conclusions of this work is that we need multiple analytical tools to perform a fair evaluation of different cell mixture deconvolution methods, and our plots (implemented in R: corrplots, cell-signature plots and bar-mixture plots) facilitate such comparative analysis. In addition, the study shows that CIBERSORT provides robust and consistent results in the deconvolution analyses of mixtures of immune cells; with high correlations in cell proportions, low RMSE values, as well as high similarity between the estimated and known cell type distributions. CIBERSORT is supervised, always using a predefined signature matrix (LM22), which includes genes that characterize the main immune cell types (as can be seen in Supplementary Figure S5). Therefore, it is necessary to define the cell types investigated, and if a sample mix includes cells that have not been predefined (e.g., cancer cells), the method does not work accurately. Other methods, such as FARDEEP (supervised), perform fairly well, but again, FARDEEP needs a well-predefined gene signature for the cell types studied. ABIS (also supervised) needs to know the true proportions in the samples, which requires prior analysis using precise experimental techniques (such as flow cytometry). For this reason, we do not recommend this deconvolution method to predict cell ratios. DECONICA (unsupervised) presents good correlations between real and estimated cell proportions, but with much higher RMSE values, indicating that it can find the trend of relative values in cellular concentrations but is not good at estimating the real proportions. Finally, LINSEED is the most successful unsupervised method, as it is very robust in the presence of noise in the data (due, for example, to the presence of unidentified cell types or contamination). Furthermore, it was also the most accurate method for optimizing the cell-specific gene signatures provided by CIBERSORT, as it was able to select more specific genes for five main types of immune cells: B-cells, T-cells, NK cells, monocytes and neutrophils. The specific gene signatures obtained in this way for each of these cell types are provided in Supplementary Figure S4.

## Figures and Tables

**Table 1:** Summary of cell mixtures datasets used in this work.

Accession Number	Gene Expression Platform	Samples	Genes	Biological Source	Cell Types	Reference
GSE64385	Microarray HGU133 Plus 2.0—*Affymetrix*	12	54,675	PBMCs ^1^, PMNs ^2^, and Cancer Cells (HCT116)	5	[10]
GSE107011	RNA-seq HiSeq 2000—*Illumina*	13	17,487	PBMCs	17	[48]
GSE106898	Microarray Human HT-12 V4.0—*Illumina*	13	17,487	PBMCs	11	[47]

^1^ PBMC: peripheral blood mononuclear cell. ^2^ PMN: polymorphonuclear cell.

## Data Availability

Data supporting the reported results can be found in the Supplementary Files provided with this article on the IJMS (MDPI) website. References to public repositories for the original human data used in this study are also provided (Table 1).

## Supplementary Materials

The supporting information can be downloaded at: https://www.mdpi.com/article/10.3390/ijms241310765/s1.
