# Supplementary material for: Comparative Analysis of Cell Mixtures Deconvolution and Gene Signatures Generated for Blood, Immune and Cancer Cells
Source: Int J Mol Sci. 2023 Jun 28;24(13):10765. doi: 10.3390/ijms241310765 (PMC10341895; doi:10.3390/ijms241310765)
Supplement: Supplementary file 1 [file ijms-24-10765-s001.zip › ijms-2392732_NAlonsoetal23_Supplementary-FIGURES-S1,S2,S3,S4,S5.pdf]

Supp. Figure S1

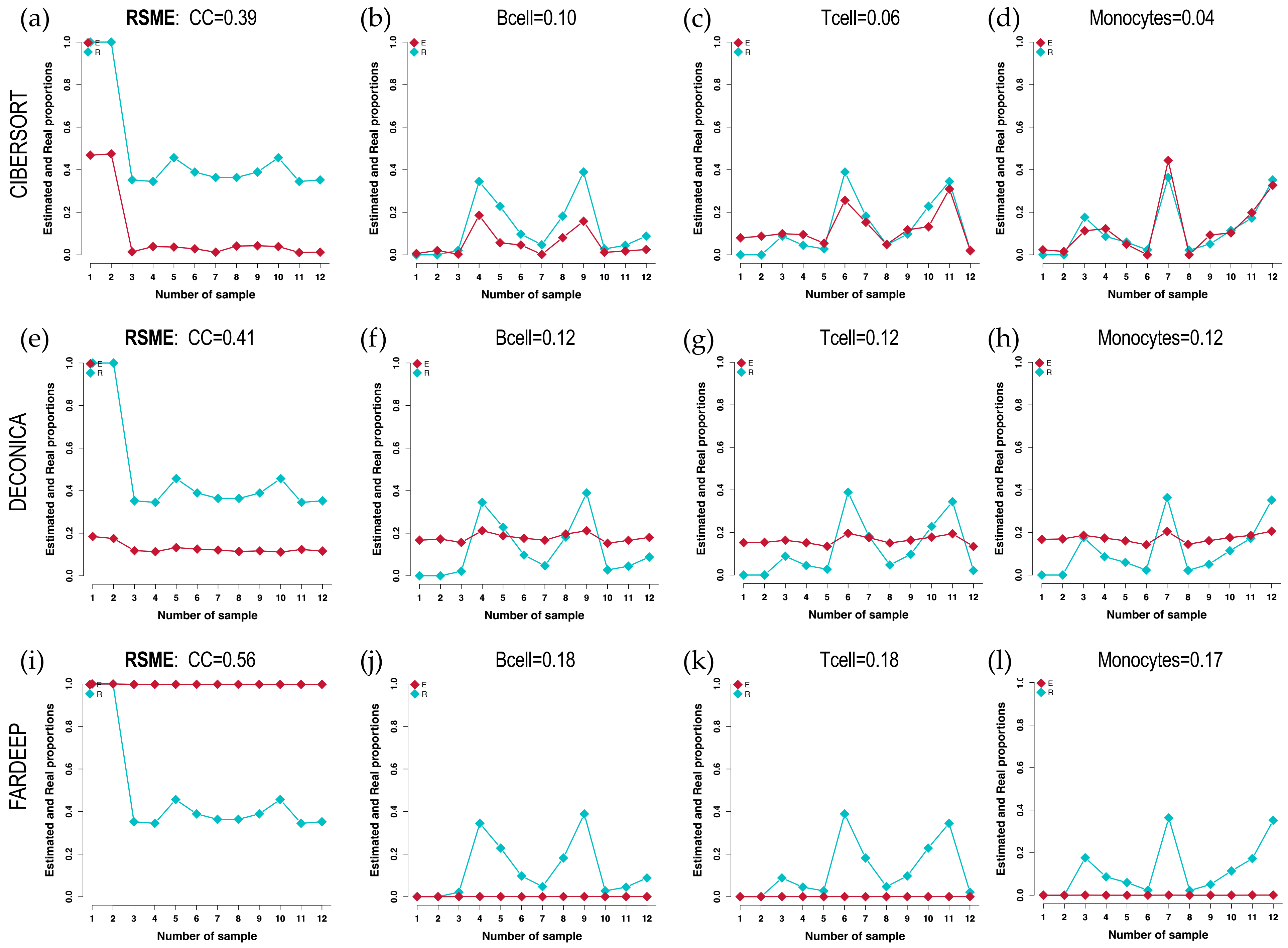

Supp. Figure S2

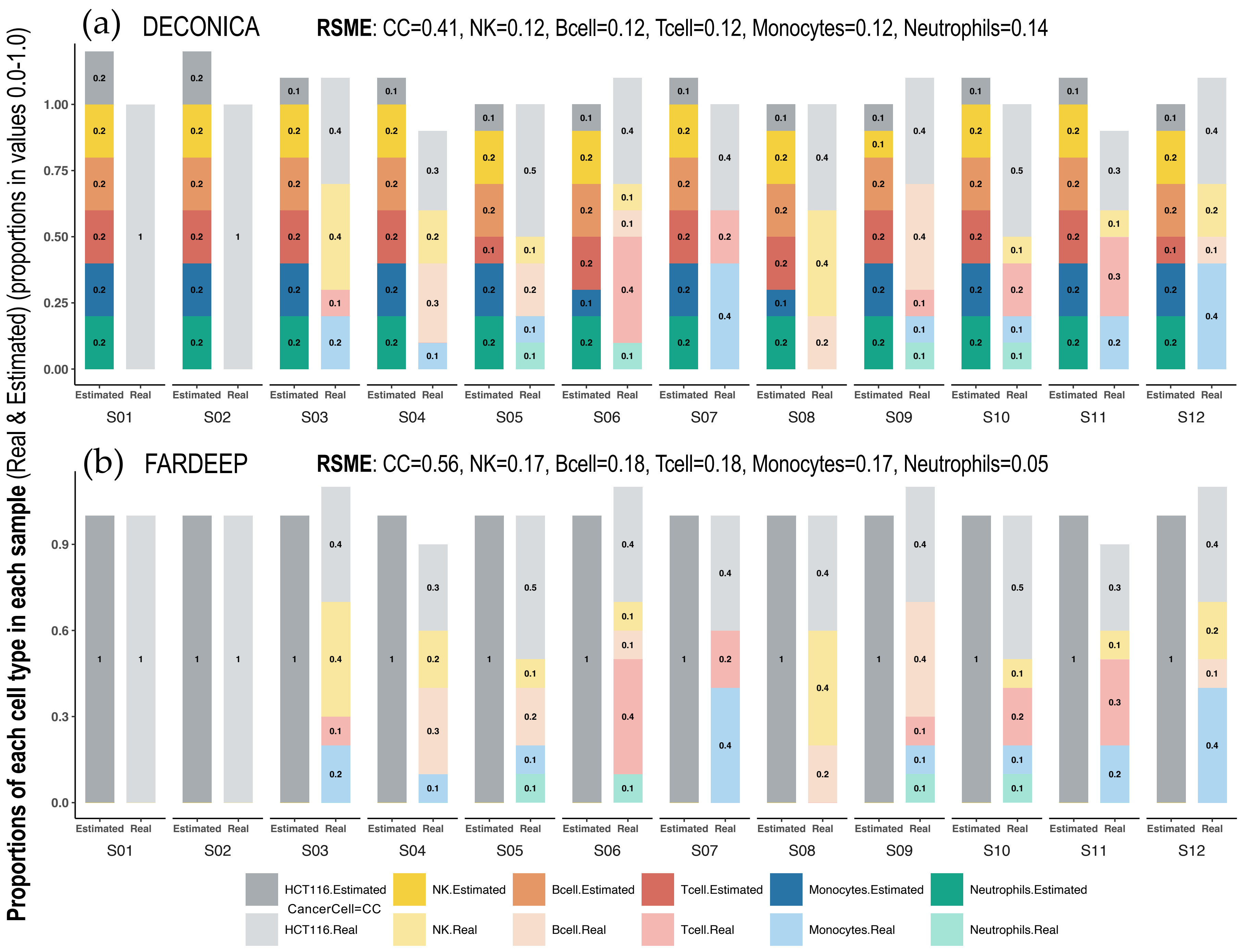

Supp. Figure S3

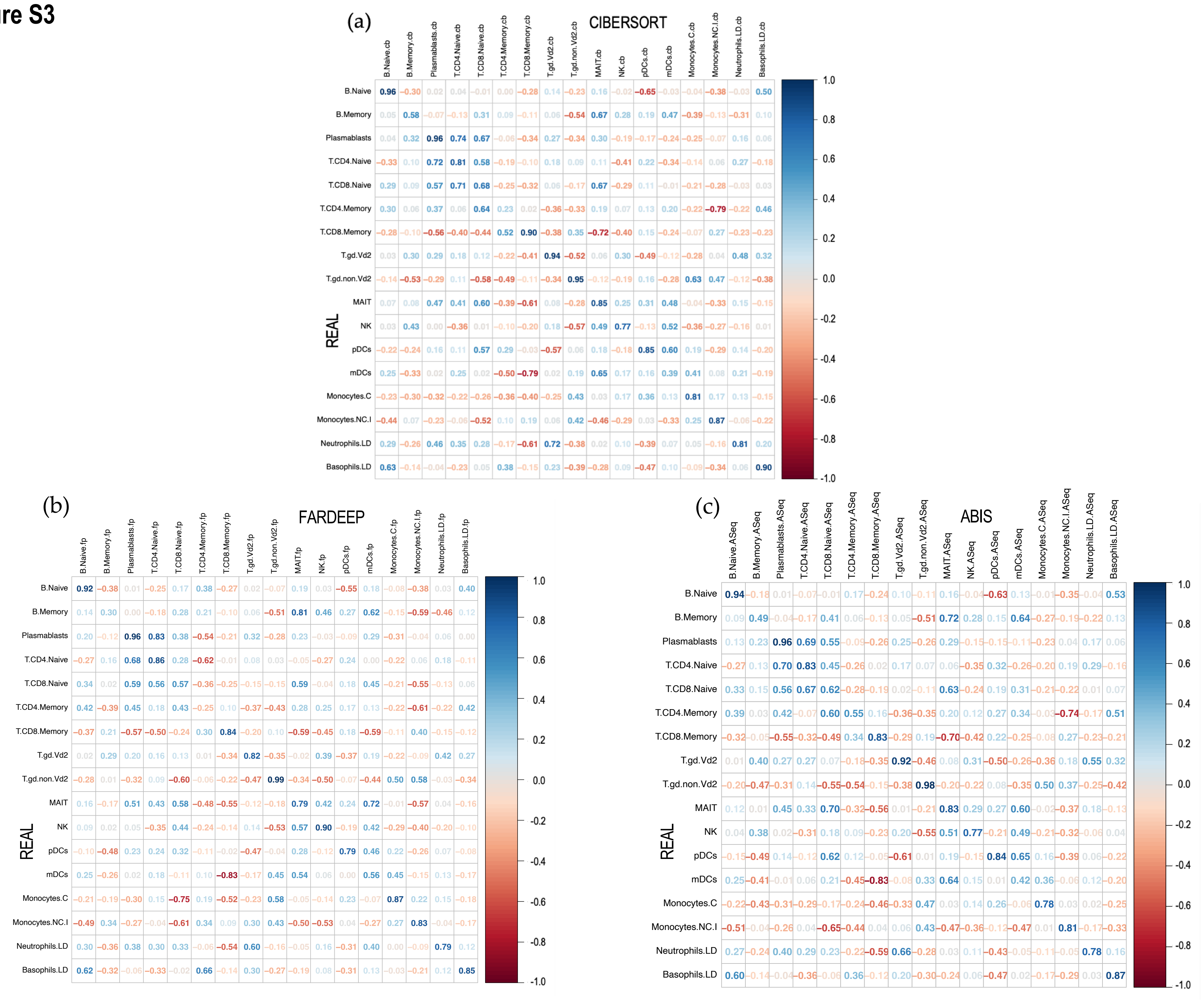

Supp. Figure S4

Cell-specific gene signatures identified by LINSEED using LM22 (from CIBERSORT)

GeneSymbol	Bcell	GeneSymbol	Tcell	GeneSymbol	NK	GeneSymbol	Monocyte	GeneSymbol	Neutrophil
ADAM28	1.7295534	ANKRD55	1.7327310	APOBEC3G	1.7832416	ASGR1	1.7682697	AQP9	1.7800075
BANK1	1.7819249	BCL11B	1.7240431	ATP8B4	1.7279334	ASGR2	1.7879561	BCL2A1	1.7650819
CD19	1.6694348	CD28	1.7535370	CCL4	1.7883725	CCR2	1.7826327	C5AR1	1.7666271
CD22	1.7836722	CD3D	1.7369996	CCL5	1.7645276	CD1D	1.7592248	C5AR2	1.7847974
CD72	1.7657127	CD3E	1.5805948	CD160	1.7792502	CD33	1.7831970	CCR3	1.7773047
CXCR5	1.7772851	CD3G	1.7070489	CD244	1.7400250	CD86	1.7611574	CHI3L1	1.7840141
FCRL2	1.7236735	CD40LG	1.7730402	CD247	1.7320778	FCN1	1.7814872	CLC	1.7152682
IGHD	1.7716216	CD5	1.7366994	CD7	1.7493547	IL1B	1.7747756	CREB5	1.7651106
IGHM	1.7678175	GPR171	1.7443490	CST7	1.7863022	MARCO	1.7594699	CXCR1	1.7620472
MS4A1	1.7733030	ICOS	1.7359114	CTSW	1.7850378	MS4A6A	1.7860920	CXCR2	1.7683026
P2RX5	1.7674058	IL7R	1.7743452	FASLG	1.7744823	RNASE2	1.7813298	DAPK2	1.7648470
PNOC	1.7677918	ITK	1.6521469	GNLY	1.7788923	11 genes signature		FCGR3B	1.7769466
RALGPS2	1.7807084	KCNA3	1.6767140	GZMA	1.7865240			FPR1	1.7576964
RASGRP3	1.7539484	LEF1	1.7770933	GZMB	1.7875933			FPR2	1.7859118
SPIB	1.7740664	SIRPG	1.7807054	GZMH	1.7849737			HAL	1.7738765
STAP1	1.6963857	TCF7	1.7422185	IL12RB2	1.7772257			HSPA6	1.5875779
TCL1A	1.7828562	TRAC	1.7798574	IL2RB	1.7846758			LILRA2	1.7269520
TMEM156	1.7477370	TRAT1	1.7820548	KIR2DL1	1.7873561			MGAM	1.7758014
TNFRSF13B	1.7499363	UBASH3A	1.7763251	KIR2DL4	1.7875498			MMP9	1.7810954
TNFRSF17	1.7423038	19 genes signature		KIR2DS4	1.7874721			MXD1	1.7379327
VPREB3	1.7793013			KLRB1	1.7559382			NPL	1.6326441
21 genes signature				KLRC3	1.7885124			P2RY13	1.5079123
				KLRC4	1.6855612			QPCT	1.7435342
				KLRD1	1.7883072			REPS2	1.7842934
				KLRF1	1.7841874			SLC12A1	1.7316956
				LAIR2	1.7832956			STEAP4	1.6856502
				NCR3	1.7798647			TNFAIP6	1.7785615
				NKG7	1.7816809			TREM1	1.7383722
				PRF1	1.7877407			TRPM6	1.7868189
				PRR5L	1.7850449			VNN1	1.5022349
				PTGDR	1.7836070			VNN2	1.7475986
				PTGER2	1.7503179			VNN3	1.7505501
				PVRIG	1.7437690			32 genes signature	
				TBX21	1.7856855				
				34 genes signature					

Supp. Figure S5

Hematological Cell Types & Subtypes tested in each dataset

Cell type family	Cell Types in Datasets GSE107011 17	GSE106898 11	GSE64385 5	Cell types in LM22 (CIBERSORT) 22
**Lymphocytes B**	B Naive	B Naive	B cells	B Naive
	B Memory	B Memory		B Memory
	Plasmablasts	Plasmablasts		Plasma cells
**Lymphocytes T**	T CD4 Naive	T Naive	T cells	T CD4 Naive
	T CD8 Naive			T CD8
	T CD8 Memory	T Memory		
	T CD4 Memory			T CD4 Memory resting
				T CD4 Memory activated
	T γδ Vd2	–	–	T γδ Vd2
	T γδ non-Vd2	–	–	–
	MAIT	–	–	–
	–	–	–	T regulatory (Tregs)
	–	–	–	T follicular helper
**Natural Killer cells**	NK	NK	NK	NK resting
				NK activated
**Dendritic Cells**	pDCs	pDCs	–	–
	mDCs	mDCs	–	DCs resting
				DCs activated
**Monocytes**	Monocytes C	Monocytes	Monocytes	Monocytes
	Monocytes NC+I			
**Granulocytes**	Neutrophils LD	Neutrophils LD	Neutrophils	Neutrophils
	Basophils LD	Basophils LD	–	–
**Eosinophils**	–	–	–	Eosinophils
**Macrophages**	–	–	–	Macrophages M0
	–	–	–	Macrophages M1
	–	–	–	Macrophages M2
**Mast Cells**	–	–	–	Mast cells resting
	–	–	–	Mast cells activated
